# The Impact of Small Molecule Binding on the Energy Landscape of the Intrinsically Disordered Protein C-Myc

**DOI:** 10.1371/journal.pone.0041070

**Published:** 2012-07-16

**Authors:** Julien Michel, Rémi Cuchillo

**Affiliations:** EastCHEM School of Chemistry, University of Edinburgh, Edinburgh, United Kingdom; Russian Academy of Sciences, Institute for Biological Instrumentation, Russian Federation

## Abstract

Intrinsically disordered proteins are attractive therapeutic targets owing to their prevalence in several diseases. Yet their lack of well-defined structure renders ligand discovery a challenging task. An intriguing example is provided by the oncoprotein c-Myc, a transcription factor that is over expressed in a broad range of cancers. Transcriptional activity of c-Myc is dependent on heterodimerization with partner protein Max. This protein-protein interaction is disrupted by the small molecule 10058-F4 (**1**), that binds to monomeric and disordered c-Myc. To rationalize the mechanism of inhibition, structural ensembles for the segment of the c-Myc domain that binds to **1** were computed in the absence and presence of the ligand using classical force fields and explicit solvent metadynamics molecular simulations. The accuracy of the computed structural ensembles was assessed by comparison of predicted and measured NMR chemical shifts. The small molecule **1** was found to perturb the composition of the apo equilibrium ensemble and to bind weakly to multiple distinct c-Myc conformations. Comparison of the apo and holo equilibrium ensembles reveals that the c-Myc conformations binding **1** are already partially formed in the apo ensemble, suggesting that **1** binds to c-Myc through an extended conformational selection mechanism. The present results have important implications for rational ligand design efforts targeting intrinsically disordered proteins.

## Introduction

It is now apparent that many proteins do not adopt a unique fold in native conditions, but rather exist as an ensemble of distinct conformations in rapid exchange. [1], [2] These intrinsically disordered proteins (IDPs) are highly abundant in nature, it has been suggested that up to half of proteins in mammals contain long consecutive stretches (>30) of disordered residues. [3] IDPs often participate in protein-protein interactions and form ordered protein-complexes by coupled folding and binding. [4] This molecular recognition mechanism is characterized by high-specificity low-affinity complexes owing to the high entropic cost of complex formation. [5] The structural flexibility of IDPs enables interactions with several protein partners, explaining why IDPs play essential roles in a broad range of cellular functions such as cell-signaling and transcription. [1], [2], [5] Additionally IDPs have been shown to be predominantly implicated in a wide range of diseases. Iakoucheva et al. report that ca. 80% of cancer-associated proteins are predicted to contain intrinsically disordered regions, [6] whereas Uversky et al. have reported ca. 60% of proteins associated with cardiovascular and neurodegenerative disorders can also be classified as IDPs. [7] Given the important role of IDPs in human health, the development of small molecule chemical probes to modulate IDP function is desirable. [8], [9] The task is challenging, historically IDPs have largely been considered “undruggable”, so there is little prior data to guide ligand-based design methods. The considerable structural flexibility of IDPs also limits the applicability of established structure-based methods such as NMR or crystallography to probe in details protein-ligand interactions. [10] Yet a few success stories suggest that small molecule inhibition of IDPs may be feasible.

**Figure 1:**
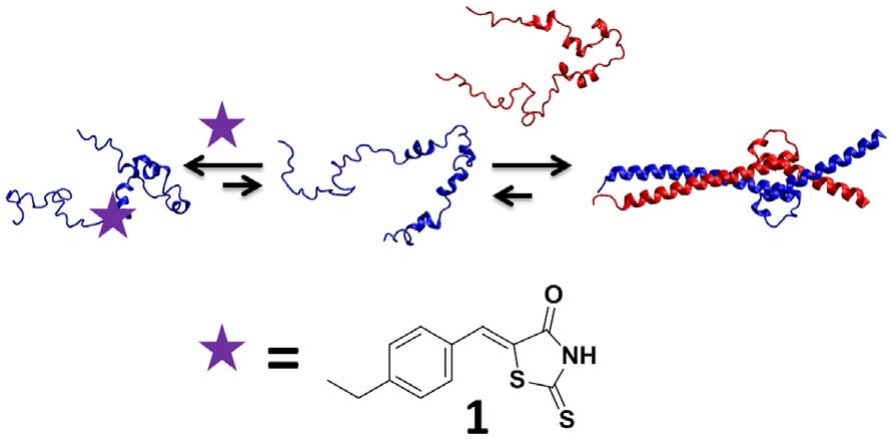
Small molecule inhibition of the c-Myc/Max interaction. The small molecule 10058-F4 (**1**, purple star) disrupts heterodimerization of the bHLHZip domains in c-Myc (blue) and Max (red) by stabilizing conformations in monomeric c-Myc incompatible with c-Myc/Max dimerizaton.

The oncoprotein c-Myc provides a striking example. Temporary inhibition of c-Myc has been shown to selectively kill mouse lung cancer cells, and c-Myc is therefore a potential cancer drug target. [11] c-Myc belongs to the Myc family of transcription factors and Myc-dependent transactivation requires heterodimerization of its basic-Helix-Loop-Helix-Leucine zipper (bHLHZip) domain with the bHLHZip domain of the partner protein Max. [12] The c-Myc/Max heterodimer interface is a parallel, left-handed, four-helix bundle where each monomer forms two α-helices separated by a small loop. The bHLHZip domains of monomeric c-Myc and Max are intrinsically disordered and the c-Myc/Max complex is thus an example of coupled folding and binding. Several inhibitors of c-Myc/Max have been identified in the past decade. [13] Notably Yin et al. used a high-throughput screen to identify structurally diverse small molecule inhibitors of the c-Myc/Max interaction. [14] Extensive biophysical studies of small molecule binding to c-Myc have been performed using NMR, circular dichroism and fluorescence assays. [15], [16], [17] These studies have led to the conclusion that many of the small molecules inhibitors disrupt the c-Myc/Max interaction by binding to monomeric c-Myc and stabilizing conformations incompatible with Max heterodimerization, as illustrated in Figure 1.

Remarkably, multiple distinct small molecule binding sites are present in the c-Myc bHLHZip domain. Hamoudeh et al. have shown that small molecule ligands that target distinct sites can bind simultaneously to the c-Myc bHLHZip domain. In addition, truncated segments of 10–40 amino acids bind different small molecule ligands with similar affinity to the full length domain. These observations suggest that the protein/ligand interactions are local and largely dictated by the protein primary sequence. [17] To illustrate, the small molecule 10058-F4 (**1**) binds c-Myc_353–437_ with a K_d_ of 5 µM and c-Myc_402–412_ with a K_d_ of 13 µM in a fluorescence polarization assay. [16] Additionally, similar chemical shift perturbations are observed upon binding of **1** to c-Myc_353–437_ and c-Myc_402–412_. Therefore c-Myc_402–412_ is a good model of the interactions of **1** with full length c-Myc. Although the measured K_d_s indicate **1** does not bind strongly c-Myc, they are similar to the measured K_d_s for formation of the c-Myc/Max complex (ca. 1 µM), [15] thus **1** can disrupt effectively the c-Myc/Max interaction. Indeed, extensive cellular studies of c-Myc function have been performed with **1**, [18], [19] but rapid clearance has hindered progress to animal models and clinical studies. [20] The development of improved c-Myc/Max inhibitors to evaluate new clinical anti-cancer therapies would benefit greatly from detailed structural models of c-Myc/small molecule interactions. [21] More generally IDPs have just started to be considered druggable and it is important to establish the mechanisms of molecular recognition between small molecules and IDPs to guide rational drug design efforts. [8], [9].

Molecular dynamics (MD) simulations present an attractive option to achieve this goal given the difficulty in obtaining high-accuracy IDP structures using biophysical methods. [10], [22] Simulation studies of IDPs do raise profound technical challenges. The reliability of standard biomolecular force fields for IDPs is not well understood as they have been historically validated against other protein classes. Further, accurate resolution of IDP ensembles requires extensive conformational sampling which remains difficult to achieve and currently requires either massive computational power, coarse-graining, implicit solvent models, or enhanced sampling methods. [23], [24] Nevertheless, molecular simulation studies have already shed new insights into IDP structure and mechanisms of interactions with other proteins. [25], [26], [27], [28], [29], [30], [31].

In the present manuscript, the bias-exchange variant of the metadynamics method (BEMD) has been used to sample extensively the energy landscape of c-Myc_402–412_ (sequence YILSVQAEEQK) and the c-Myc_402–412_/**1** complex using explicit solvent models and classical force-fields. [32], [33] Detailed comparison of the computed apo (c-Myc_402–412_ alone) and holo (c-Myc_402–412_ in the presence of **1**) structural ensembles reveals how ligand binding modulates the equilibrium ensemble of c-Myc_402–412_, provides new insights into the mechanisms of molecular recognition between a small molecule and an IDP, and has important implications for structure-based strategies to design improved c-Myc/Max inhibitors.

## Results

### Enhanced Sampling Improves the Accuracy of the Computed c-Myc_402–412_ Structural Ensemble

**Figure 2:**
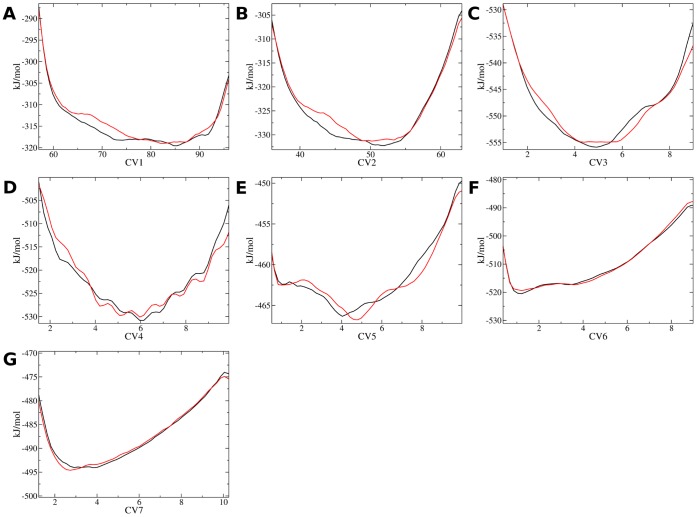
Free energy profiles for the c-Myc_402–412_ apo simulations projected along several collective variables. Black: Simulation apoA, Red: Simulation apoB. A) CV1, B) CV2, C) CV3, D) CV4 E) CV5 F) CV6 G) CV7. See the Methods section in the main text for a definition of each CV.

**Figure 3:**
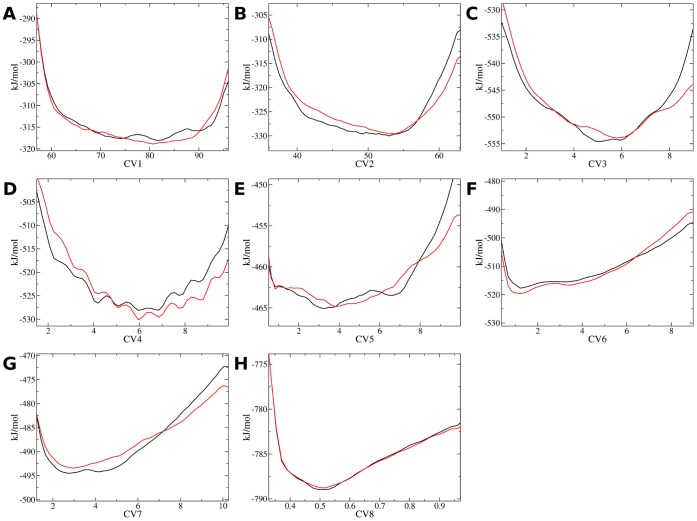
Free energy profiles for the c-Myc_402–412_/1 holo simulations projected along several collective variables. Black: Simulation holoA, Red: Simulation holoB. A) CV1, B) CV2, C) CV3, D) CV4, E) CV5, F) CV6, G) CV7, H) CV8. See the Methods section in the main text for a definition of each CV.

**Figure 4:**
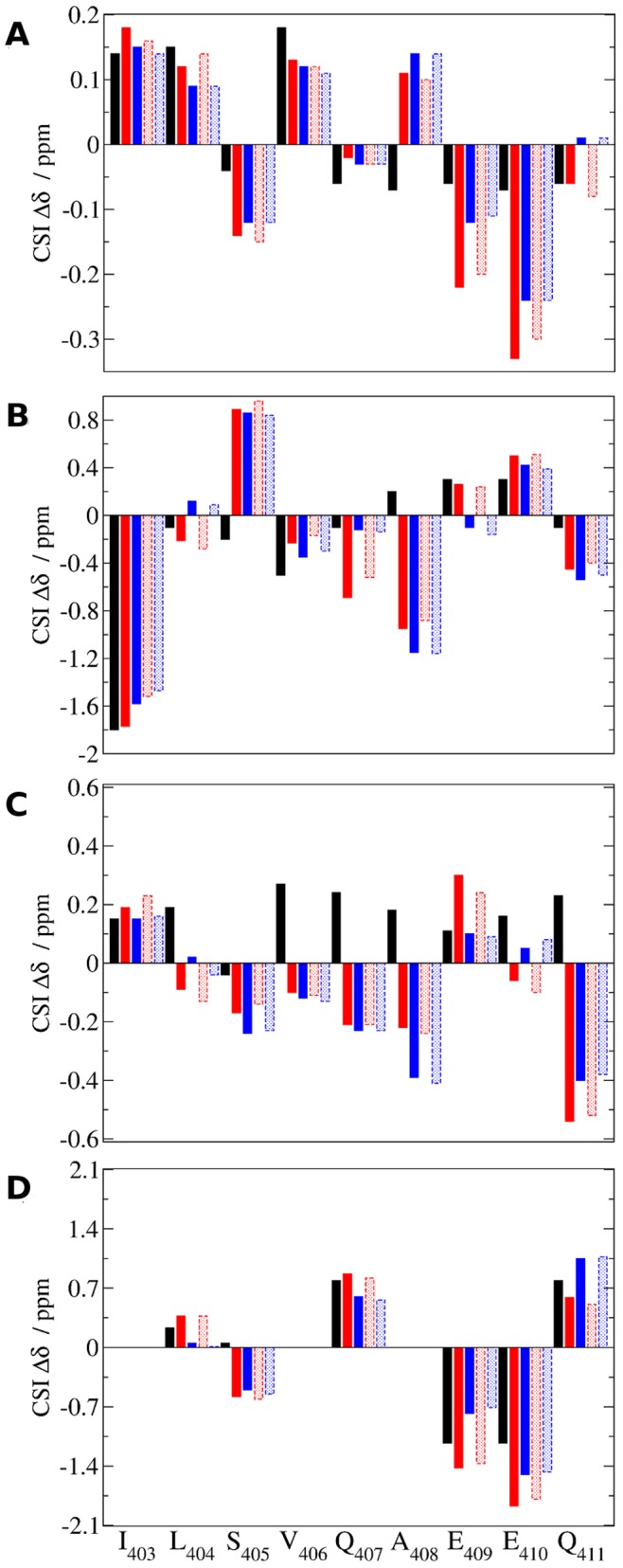
Comparison of computed and observed secondary chemical shifts for apo c-Myc_402–412_. A) ^1^H_α_ chemical shifts. B) ^13^C_α_ chemical shifts. C) ^1^H backbone amide chemical shifts. D) ^13^C_β_ chemical shifts. Black: experimental data. Solid red and blue: predicted by reweighting the biased BEMD simulations apoA and apoB respectively. Dotted red and blue: predicted from the neutral replicas of the BEMD simulations apoA and apoB respectively. Not all experimental ^13^C_β_ chemical shifts were reported. Camshift does not report chemical shifts for terminal residues.

Exhaustive enumeration of structural ensembles for IDPs is a challenging task. In this study structural ensembles for c-Myc_402–412_ and the c-Myc_402–412_/**1** complex were obtained using the bias-exchange metadynamics technique. [33] The approach entails running a set of molecular dynamics simulations. The sampling of molecular conformations in each simulation is biased by a history-dependent potential constructed as a sum of Gaussians centered on a collective variable (CV). After an equilibration period, the Gaussian biases compensate free energy barriers and rapid diffusive behavior is achieved along the CV. [34] In addition, exchanges between the biasing potentials used in the different CVs are periodically attempted according to a replica exchange scheme. Finally, a neutral replica that is not biased by any CV is also simulated. The neutral replica has been shown in other studies to produce an ensemble similar to the equilibrium ensemble of the system. [33], [35], [36], [37] BEMD has been shown to allow extensive sampling of the folding free energy landscape of small proteins and protein/ligand complexes on timescales of a few dozen ns (see Methods for details on the protocols used). [32], [38] Convergence of the simulations was first assessed by constructing several one dimensional free energy profiles along the CVs used to enhance conformational sampling. These were obtained from the negative of the accumulated Gaussian biases along each CV, which in the limit of a sufficiently long simulation, reproduce the free energy of the system up to an additive constant. To assess reproducibility of the free energy profiles two apo and holo simulations were performed, each initiated from uncorrelated sets of configurations. The results are shown in Figure 2 and 3. In general the free energy profiles within 10 kJ/mol of the global minimum are well reproduced (within ca. 1 k_B_T or less) for most CVs between the two independent simulations. The largest discrepancy is observed for CV2 in the apo simulations in the range of CV values of 40–50. In the holo simulations, more variability is observed in the regions of high free energy for CV3 and CV4 (Fig. 3C and Fig. 3D). Conformations in these regions contribute little weight to the equilibrium ensemble, and the overall equilibrium properties obtained by reweighting statistics from the biased simulations (see Methods) are fairly consistent for the two independent simulations (e.g. Table 1 and Figure 4). Visualization of the apo neutral replica ensemble reveals that a broad range of compact, extended, structured and unstructured conformations have been sampled in both simulations (Figure S1 panel A). The BEMD neutral replica ensembles were compared to two 100 ns unbiased MD simulation performed using the same potential energy function and system setup. The first MD simulation was initiated from an extended conformation which collapses into a short α-helical conformation around Leu_404_-Ala_408_ after a few ns. This α-helix occasionally briefly unwinds, but remains otherwise stable throughout the simulation, eventually extending to include Ile_403_ and Tyr_402_ after about 90 ns (Figure S1 panel C). The conformations sampled in the second MD simulation are very different and mostly unstructured (Figure S1 panel D). Thus the unbiased MD simulations provide an inconsistent picture of the c-Myc_402–412_ structural ensemble in comparison to the BEMD simulations. It is likely that orders of magnitude increase in the duration of the MD simulation would be necessary to achieve a sampling of the energy landscape comparable to the BEMD simulation for this system.

**Table 1:** Percentage of secondary structure content of apo c-Myc_402–412_.^1^

	Helix		Sheet		Polyproline II
**BEMD run 1**	13.9	10.0	0.7	0.9	12.6
**BEMD run 2**	10.9	9.4	0.6	0.2	12.1
**BEMD run1 neutral**	14.2	11.9	0.7	1.0	11.6
**BEMD run2 neutral**	10.5	9.2	0.5	0.2	11.7
**MD run 1**	34.3	41.5	0	0	5.5
**MD run2**	0.1	0.1	0.5	0.5	13.5
**Exp** 2	4		3		13

1 helix and sheet content computed according to the DSSP and STRIDE methods respectively. A helix was defined as G + H + I and a sheet as B + E using the 7 letter DSSP code. The polyproline II content was estimated using the software PROSS.

2 Secondary structure content estimated from measured chemical shifts, using the webserver δ2d.

To assess the accuracy of the computed ensembles, snapshots collected during the MD and BEMD simulations of c-Myc_402–412_ were used to back-compute NMR chemical shifts using the software Camshift. [39] Figure 4 compares with available experimental data the computed ^1^H and ^13^C secondary chemical shifts for H_α_ protons, backbone amide protons, C_α_ and C_β_ carbons. [15] The secondary chemical shifts computed from the two BEMD ensembles are fairly consistent and there is little difference between the chemical shifts obtained by averaging over snapshots from the neutral replicas or by reweighting snapshots from the biased simulations (Figure 4). By contrast, greater variability and inconsistency is observed between the chemical shifts computed from the two unbiased MD simulations (Figure S2). The mean-unsigned errors for the H_α_, H, C_α_ and C_β_ chemical shifts computed from the two reweighted BEMD simulations are: 0.09/0.08, 0.43/0.44, 0.32/0.28 and 0.35/0.32 ppm respectively. These figures are very similar to the mean-unsigned errors computed for the neutral replica ensembles: 0.09/0.08, 0.45/0.46, 0.32/0.29 and 0.34/0.35 ppm respectively. By comparison the mean-unsigned errors computed from the two MD simulations are: 0.15/0.13, 1.25/0.80, 0.50/0.42, 1.01/0.86 ppm respectively. Thus in addition to predicting equilibrium properties that are much more consistent between independent runs, the overall errors in predicted secondary chemical shifts have been roughly halved using the BEMD protocol.

The overall secondary structure content of the computed ensembles estimated using the software DSSP, [40] STRIDE, [41] and PROSS [42] for the BEMD and MD simulations is reported in Table 1. These figures can be compared to the secondary structure content estimated from experimentally measured chemical shifts using the software δ2D. [43] The first MD ensemble largely overestimates the helical content of c-Myc_402–412_, fails to detect any sheet content and underestimates the polyproline II content. The second MD ensemble has a negligible amount of helical structures or sheet content, but reproduces better the polyproline II content measured experimentally. By contrast the structural ensembles computed by BEMD simulations are fairly consistent to within a few percent. The helical content is overestimated and the sheet content slightly underestimated, which may reflect a systematic bias from the force field used in the simulations. The computed polyproline II content is otherwise in good agreement with the measured polyproline II content. Given that both simulations have been performed on the same system with the same force field, the greater discrepancies in computed observables for the MD simulation arise from greater sampling errors. Although it is likely that optimized force fields could decrease further discrepancies with experiment, the computed BEMD ensemble is overall in reasonable agreement with the available experimental data for this system.

The equilibrium properties of c-Myc_402–412_ predicted by reweighting the biased simulations or by simple averaging over snapshots sampled by the neutral replica are remarkably similar (Table 1, Fig. 4). This suggests, in agreement with other bias-exchange metadynamics studies, that the neutral replica is a good approximation of the canonical ensemble. To assert further this claim, one-dimensional free energy profiles along all collective variables used in the biased simulations were constructed from the statistics collected in one of the neutral replica. Comparison of the free energy profiles between the neutral replica and the biased simulations (Figure S3) indicates that the global minimum in free energy is well reproduced, but that regions of high free energy are systematically overrepresented in the neutral replica. This observation explains why the equilibrium properties predicted by reweighting the biased simulations or by averaging over snapshots sampled from the neutral replica agree well, since conformations of low free energy contribute with a greater weight to the equilibrium properties of the system. Nevertheless this analysis suggests that the accuracy of the neutral replica ensemble decreases rapidly for conformations of higher free energy. Consequently, analyses in the rest of the manuscript were performed on ensembles constructed by reweighting snapshots from the biased simulations (see Methods).

### The c-Myc_402–412_ Apo Ensemble Contains Collapsed, Extended and Helical Conformations

**Figure 5:**
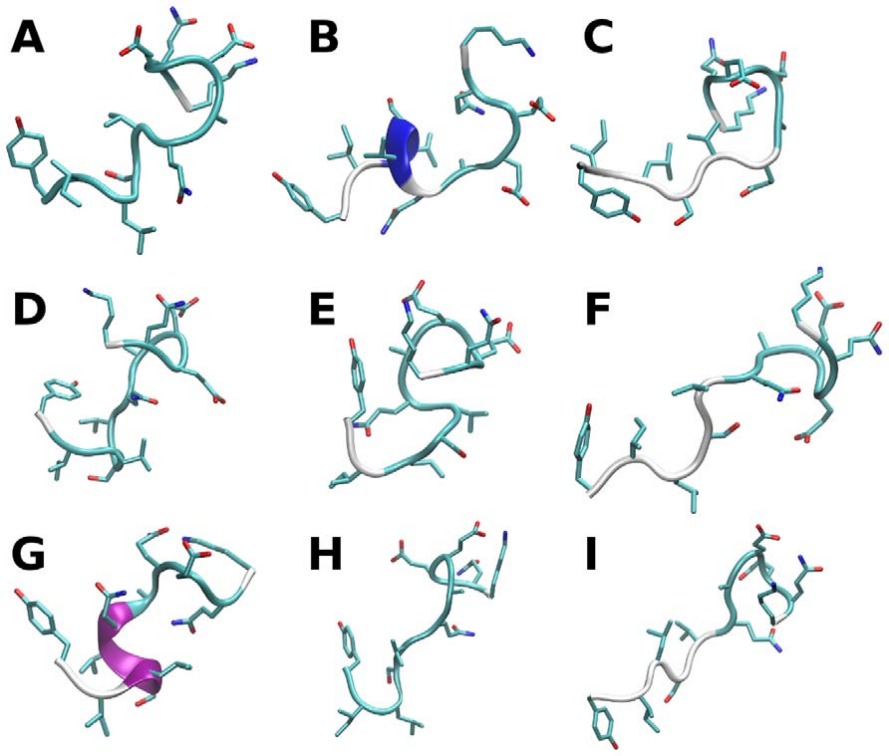
Representative conformations from the computed equilibrium ensemble for apo c-Myc_402–412_. The conformations depicted are those closest to the center of the most populated clusters. The fractional cluster populations are: 0.101±0.018 (A), 0.075±0.034 (B), 0.060±0.040 (C), 0.059±0.027 (D), 0.055±0.016 (E), 0.043±0.004 (F), 0.030±0.017 (G), 0.021±0.009 (H), 0.021±0.003 (I). Figure prepared with the software VMD [78].

The equilibrium ensemble is heterogeneous and includes several extended and collapsed conformations, consistent with c-Myc_402–412_ being intrinsically disordered. Clustering indicates there are dozens of structurally diverse clusters of conformations. [44] Figure 5 shows representative conformations from the ninth largest clusters calculated for the apo ensemble. Although the ensemble properties of the peptide are well reproduced, not all clusters are equally well populated in the two independent BEMD simulations, as evidenced by the standard error estimates of the cluster populations. Longer simulations would be required to obtain more precise population estimates. The largest cluster (Figure 5A) is a random coil structure stabilized by hydrophobic contacts between Tyr_402_, Ile_403_ and Val_406_ and electrostatic interactions between Lys_412_ and Glu_409_. Other partially collapsed conformations are apparent (e.g. Figure 5D and 5E), but extended conformations are also observed (e.g Figure 5F and 5I). Additionally, several clusters include conformations containing short α or 3_10_ helices (Figure 5B, 5G), that account for the overall computed helical content of c-Myc_402–412_.

### c-Myc_402–412_ Remains Disordered upon Binding the Small Molecule 10058-F4

**Figure 6:**
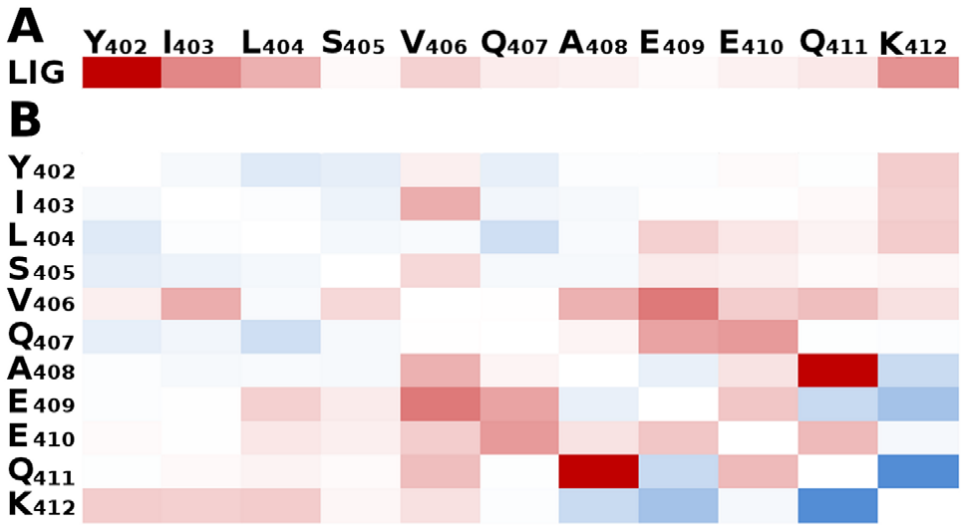
Average number of contacts between 1 and c-Myc_402–412_. A) Average number of ^1^H contacts between different c-Myc_402–412_ residues and **1.** Color coded from white (no contacts) to red (high number of contacts). The extreme values of this color scale range from 0.02 to 1.08. B) Difference in the average number of ^1^H contacts between different c-Myc_402–412_ residues in the holo and apo ensembles. Red/blue indicates an increased/decreased average number of contacts upon binding of **1**. The extreme values of this color scale range from −1.22 to +0.67.

To assess the impact of the binding of **1** on the conformations of c-Myc_402–412_, the average number of contacts between protons (*d*<3.0 Å) in **1** and different protein residues was computed for the apo and holo BEMD simulations. Figure 6 shows the difference in contact probabilities between the holo and apo simulations, red indicates increased contact probabilities, blue indicates decreased contact probabilities, whereas white indicates unchanged contact probabilities. Figure 6A shows that **1** contacts primarily the N-terminal region of c-Myc_402–412_, with a strong preference for contacts with Tyr_402_. Lys_412_ is the only side-chain in the C-terminal region of c-Myc_402–412_ that forms significant contacts with **1**. Given that **1** contains a moderately polar heterocycle and a hydrophobic ethylphenyl group, it is not surprising that intermolecular contacts occur preferentially with the N-terminal region as it is enriched in hydrophobic amino acids. Figure 6B depicts the difference in average number of contacts between protein residues in the apo and holo simulations. This analysis reveals whether ligand binding changes contact probabilities between residues in c-Myc_402–412_. Overall decreased contacts of Tyr_402_ with nearby amino-acids are observed because **1** lies frequently between these side-chains. Increased contacts between Lys_412_ and the N-terminal amino acids correlate with decreased contats with amino acids in the C-terminal region. This occurs because c-Myc_402–412_ adopts more frequently conformations that wrap around **1**. Comparison of computed and measured chemical shifts for the c-Myc_402–412_/**1** complex is not possible owing to the lack of parameters in Camshift to describe the ligand. However the simulations suggest formation of a hydrophobic cluster between **1** and the side chains of Tyr_402_, Ile_403_, Leu_404_, Val_406_, which is in qualitative agreement with the interpretation of the limited NOEs measured by Follis et al. [16] The holo ensemble remains heterogeneous, thus binding of **1** does not structure considerably c-Myc_402–412_. The overall helical content, 13±1% or 11±1% (DSSP and STRIDE respectively), is relatively unchanged from the apo ensemble. The negligible sheet content, and polyproline II content, 12±1% (PROSS) are broadly similar to the quantities computed for the apo ensemble.

### The Small Molecule 10058-F4 Binds to Multiple Distinct c-Myc_402–412_ Conformations

**Figure 7:**
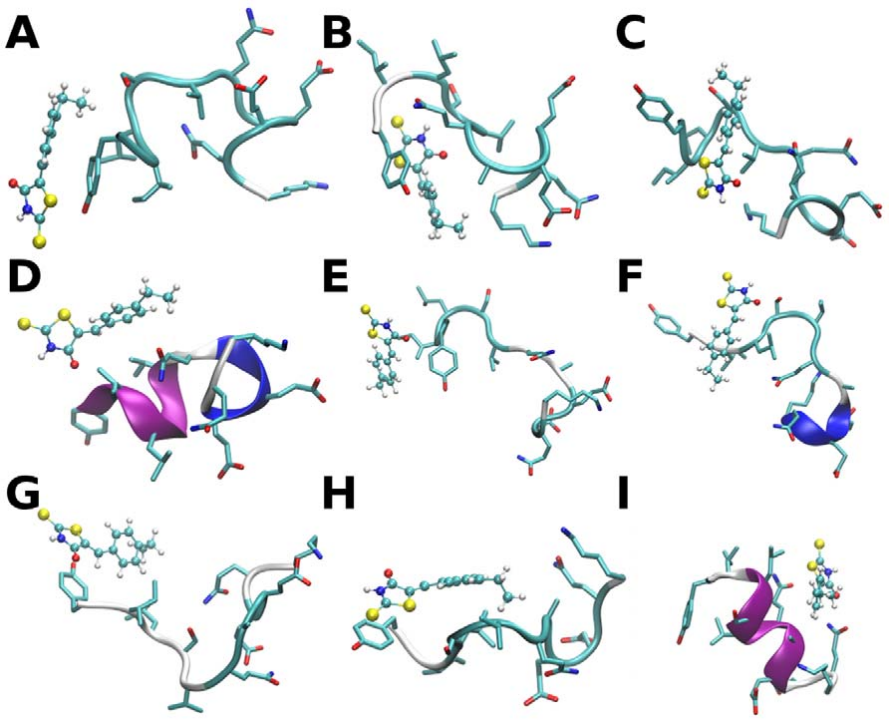
Representative conformations from the computed equilibrium ensemble for the c-Myc_402–412_/1 complex. The conformations depicted are those closest to the cluster center. The fractional cluster populations are: 0.021±0.008 (A), 0.019±0.002 (B), 0.018±0.005 (C), 0.015±0.010 (D), 0.014±0.003 (E), 0.011±0.008 (F), 0.011±0.005 (G), 0.011±0.001 (H), 0.010±0.003 (I). Figure prepared with the software VMD [78].

Although there are no dramatic changes in secondary structure content upon ligand binding, detailed analysis reveals that the nature and the population of the c-Myc_402–412_ equilibrium conformations is substantially affected by **1**. The ligand retains considerable mobility and can adopt a multitude of different binding modes against structurally distinct c-Myc_402–412_ conformations. Consequently clustering analysis of the holo ensemble produces a large number of negligibly populated clusters. Nevertheless, it is possible to identify more frequently observed binding modes. Figure 7 depicts nine conformations representative of the most populated clusters from the holo ensemble. The first cluster (Figure 7A) features stacking interactions between the phenyl rings of **1** and Tyr_402_, as well as hydrophobic contacts between the ethylphenyl group of **1** and Leu_404_. Ile_403_ forms a small hydrophobic cluster with Leu_404_ and Tyr_402_. Additional stabilizing hydrogen-bonding interactions between Gln_411_ and the peptide backbone are present. In Figure 7B the ethylphenyl group of **1** is sandwiched between the side-chains of Tyr_402_ and Lys_412_, whereas the thiazolidinone ring forms hydrogen bonding interactions with the backbone of Leu_404_ and Gln_407_. In some cases (Figure 7D, 7I) the ligand stabilizes α-helical conformations whereas in several other clusters, **1** forms relatively limited contacts with the more hydrophobic N-terminus (Figure 7E, 7F, 7G, 7H). Comparison of the computed holo c-Myc_402–412_ conformations with the conformation of c-Myc_402–412_ observed in the crystallographic structure of the c-Myc/Max dimer systematically indicates steric clashes with Max, [12] thus binding of **1** to c-Myc is not compatible with c-Myc/Max dimerization.

The broad range of c-Myc conformations binding **1** may explain the relatively forgiving structure-activity relationships observed for analogs of **1**, [21], [45] i.e. few small ligand modifications would prevent binding to all observed conformations. The most populated conformations do not closely resemble the structure of the c-Myc_402–412_/**1** complex derived using chemical-shift constraints and docking. [16] As it has been pointed out by Follis et al., a single average structure obtained from minimization of NMR derived restraints may not be representative of the multiple distinct conformations adopted by a disordered protein. [16] This highlights the usefulness of molecular dynamics simulation protocols to generate structural ensembles for IDPs and guide the interpretation of NMR measurements.

### The c-Myc_402–412_ Conformations Binding 10058-F4 are Partially Formed in the Apo Ensemble

**Figure 8:**
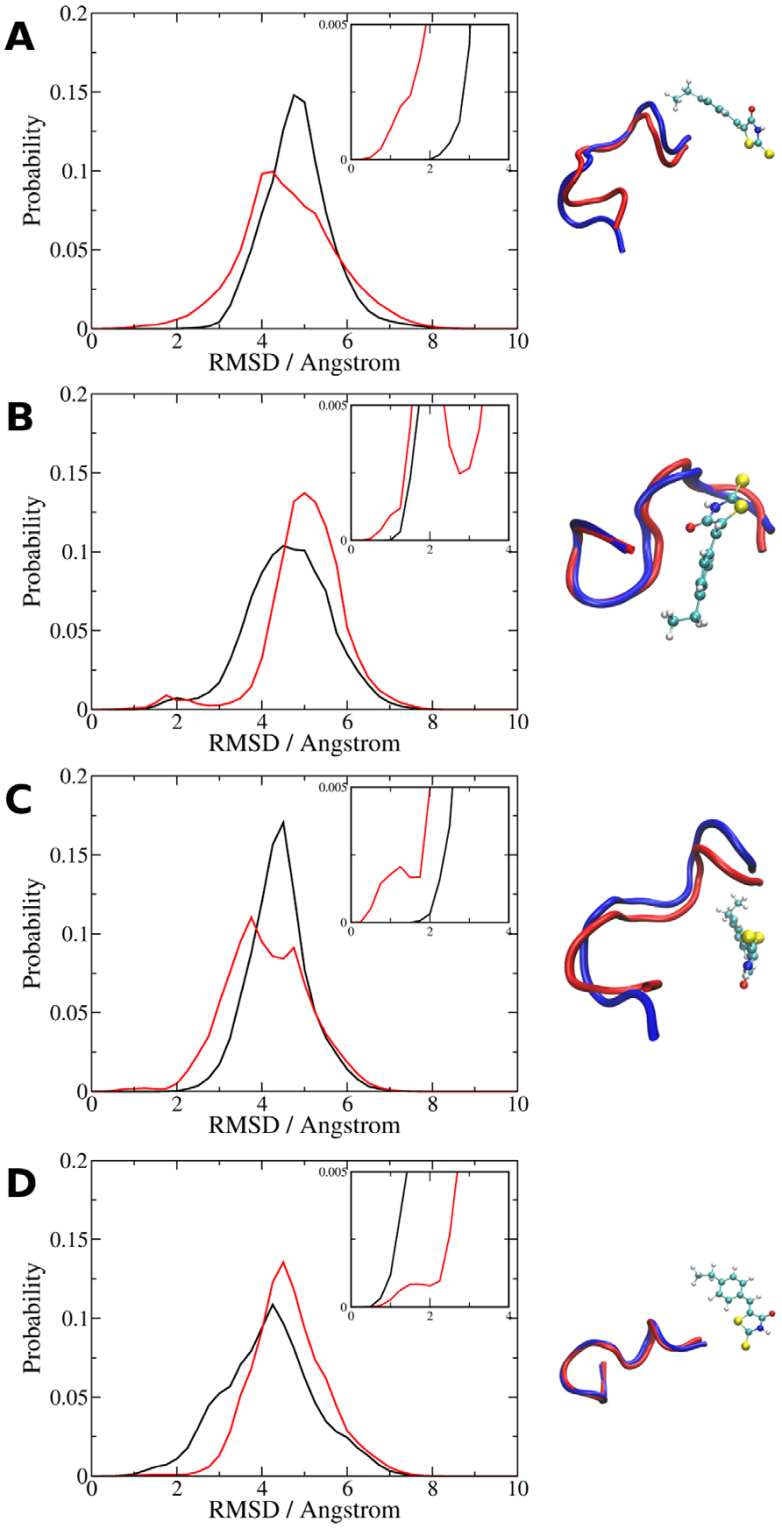
Comparison of selected holo and apo conformations to the apo and holo ensembles. A) Probability distribution of backbone RMSD of conformations from the apo (black curve) and holo (red curve) ensembles to: A) holo cluster center 7A, B) holo cluster center 7B, C) holo cluster center 7C, D) apo cluster center 5A. The inset shows the low-RMSD regions. Each panel also shows an overlay of the lowest RMSD apo or holo structure to cluster centers from panels A–D. For clarity only the peptide backbone (tube representation, apo conformations in blue, holo conformations in orange) and ligand atoms (CPK) are shown. Figure prepared using VMD [78].

To investigate the mechanisms of molecular recognition, the frequently observed apo and holo c-Myc_402–412_ conformations were compared to the computed apo and holo ensembles. Broad fluctuations in backbone conformations are observed within the apo and holo ensembles, Figure 8A–D reports histograms of backbone root mean square deviation (RMSD) of the apo and holo structural ensembles to selected holo and apo conformations depicted in Figure 5 and Figure 7. There is some arbitrariness in the definition of a criterion to consider whether two conformations are structurally similar, but overlay of several low RMSD structures suggests that a backbone RMSD around 2.5 Å or less identifies broadly similar backbone conformations for this system. According to this criterion, in all cases the c-Myc_402–412_ apo ensemble contain conformations that are structurally similar to those seen more frequently in the holo ensemble (Figure 8A–8C, insets), albeit with a lower probability. Likewise, the holo ensemble also contains conformations that have a RMSD <2.5 Å to the apo conformation shown in Figure 5A (Figure 8D). To illustrate, Figure 8 also depicts an overlay of the conformation sampled from the apo (Figure 8A–8C) or holo (Figure 8D) ensemble that has the lowest RMSD to the apo/holo conformations depicted in Figure 7A–7C and Figure 5A. These results suggest that there is significant structural overlap between the apo and holo ensembles. However additional side-chain adjustments are typically necessary to dock **1** into the apo structures to reproduce the holo conformations depicted in Figure 7 because a few side-chains would otherwise clash with the ligand.

## Discussion

In comparison with standard molecular dynamics, the present bias-exchange metadynamics simulations have been shown to dramatically enhance conformational sampling in explicit solvent of a segment of the intrinsically disordered protein c-Myc. These results add to the growing literature evidence for the usefulness of metadynamics to study biomolecular interactions. [32], [38] Because standard biomolecular force fields have not been tested extensively on intrinsically disordered proteins, it is important to validate as much as possible computed trajectories against experimental data. NMR is an established methodology to perform experimental studies of IDPs structure, and the back-computation of chemical shifts from molecular dynamics trajectories provides an excellent opportunity to connect simulations with experiments for disordered proteins. [46] The larger errors in predicted chemical shifts for the MD simulation versus the BEMD simulations reported in this manuscript highlight the importance of achieving a broad sampling of energy landscapes, at least the regions of low free energy, to reliably compare simulations with experiments. The simulated ensembles will otherwise not be well reproducible, and it will be difficult to diagnose systematic force field errors and devise more accurate potential energy functions [47].

The current results indicate that there is large conformational heterogeneity in both the apo and holo equilibrium ensembles of c-Myc_402–412_. To obtain a complete picture of the energy landscape it would be desirable to obtain as well information about kinetic properties. Owing to the use of a replica-exchange methodology and a time-dependent biasing potential, this information is not readily accessible from the present BEMD simulations. It is possible to project the BEMD trajectories on a space defined by the collective variables to build a kinetic model, [35] however we find that this analysis is of limited utility here because the structural ensemble of c-Myc_402–412_ is too diverse to resolve well several kinetic basins in a low dimensional CV space. An interesting alternative would be to conduct extended unbiased MD simulations to reversibly simulate binding/unbinding in this system and analyze the computed trajectories using Markov State models. [48], [49] Such study could also allow in principle a direct estimation of dissociation constants and kinetics of binding and conformational transitions, [50] although it may be difficult to unambiguously define bound/unbound states for an IDP.

An intriguing result from this study is the observation that no clearly dominant binding mode emerges for the c-Myc_402–412_/**1** complex. Rather, binding seems to result from a multitude of weak interactions to distinct conformations. This contrasts with the frequently observed disorder-to-order transitions in complexes involving one or two IDPs. [5], [51] Coupled folding and binding typically arises from a fine balance between a large conformational entropy loss and the formation of several intermolecular contacts across an often large interface. [5] Arguably, a drug-like molecule may be too small to form extended contacts that could overcome the large conformational entropy loss required to structure a disordered protein. Thus it seems more likely that the current small molecule inhibitors of c-Myc/Max stabilize a broad range of inactive c-Myc conformations, rather than conformationally trapping c-Myc in an inactive state. [52] The fact that several structurally diverse small molecules inhibitors of c-Myc/Max were identified with a reasonable success rate through screening relatively modest libraries supports the hypothesis that the conformational flexibility of IDPs facilitates interactions with small molecules through a large number of weak interactions. [8] Further evidence in support of this molecular recognition mechanism is provided by examples of IDPs that remain partially disordered when in complex with other proteins, for instance the CFTR/NBD1 complex, [53] or the cytoplasmic domain of the T-cell receptor ε chain/SIV nef protein complex. [54] The lock and key model cannot explain binding in the system studied here, but neither would pure conformational selection or induced fit mechanisms. The backbone conformations of c-Myc_402–412_ that more frequently bind **1** are populated with a lower probability in the apo ensemble, but further minor conformational adjustments, mainly repositioning of side-chains, are necessary before **1** can be docked without steric clashes. These observations are more in line with the extended conformational selection model to describe ligand binding in this system. [55] A recent simulation study using Gõ models from Ganguly et al. also favors this mechanism for the binding of the NCBD domain of CBP with the p160 steroid receptor coactivator ACTR. [56] Cino et al. have also reached similar conclusions from a molecular dynamics study of the interactions of the IDP Prothymosin alpha with the Neh2 domain of Nuclear factor erythroid 2-related factor 2 and Kelch-like ECH-associated protein 1. [57] To obtain further evidence, extensive reversible atomistic simulations of the binding/unbinding of **1** to c-Myc_402–412_ are desirable to establish the nature of the transition state conformations.

The broad range of observed protein/ligand interactions in this system raises profound questions regarding the possibility of designing specific small molecule ligands for IDPs. The contact matrix in Figure 6A, as well as the representative snapshots in Figure 7 suggest that **1** interact preferentially with Tyr_402_. The primary sequence of the c-Myc bHLHZip domain contains a single Tyrosine, furthermore, the c-Myc segment 401–406 contains a cluster a hydrophobic amino acids that defines the most hydrophobic region of the c-Myc bHLHZip sequence in a hydrophobicity plot (Figure S4). Thus a simple explanation for the location of the c-Myc binding site of **1**, is that the ligand binds to this segment because it contains several hydrophobic and aromatic residues. Interestingly, the bHLHZip domain of the protein Max lacks such hydrophobic clusters and appears overall less hydrophobic on a hydrophobicity plot (Figure S4). This may explain why the ligand **1** does not disrupt the Max/Max homodimer. Nevertheless, experimental evidence suggests that many of the small molecules inhibitors of c-Myc/Max identified from *in vitro* and cellular assays also disrupt other related protein-protein interactions. [8] In a series of yeast two hybrid assays on 32 HLH, HLHZip or bZip pairs, the small molecule **1** was found to inhibit strongly c-Myc/Max, but also to inhibit moderately Myod/E2–2, Mad1/Max, Mxi1/Max and Mad3/Max. [14] Given that the present simulations suggest that ligand binding to c-Myc is primarily driven by weak non specific interactions with hydrophobic patches, it is interesting to establish why **1** has been identified as a c-Myc/Max inhibitor in previous high-throughput screens. A noteworthy feature of **1**, as well as several other reported c-Myc ligands, is the presence of a benzylidene rhodanine scaffold. Such class of small molecules frequently produces low-micromolar hits in a broad range of assays and against diverse biomolecular targets. [58] Mengden et al. have analysed in details the binding promiscuity of rhodanines and concluded that this behavior is not related to aggregation or reactivity towards biological nucleophiles, but rather that this scaffold has a pronounced propensity to form intermolecular interactions with proteins. [59] Indeed Wang et al. recently reported the discovery of a benzylidene-rhodanine ligand highly similar to **1** (o-nitro group instead of p-ethyl group on the benzylidene group) and that binds to the bZIP domain of the transcription factor ΔFosB. [60] Although other scaffolds have been reported to disrupt the c-Myc/Max interaction by binding to monomeric c-Myc, these observations suggest that the binding specificity of novel c-Myc ligands should be carefully assessed against a broad range of targets. There are documented cases of initial non specific hits obtained from small molecule library screen that were subsequently optimized to show higher specificity for c-Myc/Max. [61], [62] Although clearly a challenging endeavor, one could seek to exploit the present computational approach to modify **1** in order to enhance binding affinity towards c-Myc conformations that offer several contacts to the ligand whilst minimizing binding affinity to conformations likely to offer little ligand specificity. Alternatively, larger synthetic molecules that disrupt c-Myc/Max by folding c-Myc upon binding may achieve higher binding specificity.

## Materials and Methods

### Metadynamics Simulations

The AMBER99SB^*^ forcefield was selected for c-Myc_402–412_ as it has been calibrated to reproduce the secondary structure preferences of peptides, [63] the GAFF forcefield for **1**, [64] and the TIP3P model was used for water. [65] The GAFF parameters for the ligand were obtained by using the software acpype, [66] in combination with the antechamber utility from the AMBER11 software package. [67] Atomic partial charges were assigned using the AM1-BCC method. [68], [69] Molecular models of c-Myc_402–412_ and the c-Myc_402–412_/**1** complex were built in an extended conformation using the software Maestro. [70] The peptide termini were acetylated and amidated to be consistent with experimental data. The models were then solvated in a triclinic box of 2843 and 3211 water molecules respectively and charge neutrality was enforced through introduction of one sodium ion.

The simulations were performed at 300 K and 1 atm with the software package GROMACS 4.5.5, [71] compiled with the metadynamics plugin PLUMED 1.3. [72] Apo and holo simulations were initially equilibrated in NPT conditions, using a stochastic Berendsen thermostat and Parinello-Rahman barostat with relaxation times of 0.1 and 2 ps respectively. [73], [74] A time step of 2 fs was used. Particle-mesh Ewald was used to treat long-range electrostatic interactions with a short-range cutoff of 0.9 nm. A cutoff of 0.9 nm was used for the Lennard-Jones interactions. A long-range correction term was used for the energy and pressure. [75] After NPT equilibration, BEMD simulations were performed in NVT conditions as the PLUMED software does not compute the contribution of the metadynamics forces to the virial. Short preliminary runs were performed to optimize the selection of CVs and Gaussian parameters. The CVs were chosen on the basis of previously published BEMD studies to remove possible energetic barriers between degrees of freedom describing backbone and side-chain conformational changes. The parameters of the CVs (Gaussian height and width), which control the rate of convergence and accuracy of the free energy profiles were adjusted in preliminary runs in implicit solvent so as to obtain reasonably converged free energy profiles on a timescale of several dozen nanoseconds. The apo and holo simulations were performed with 8 and 9 replicas respectively. In the production runs, each replica was simulated for 120 ns. Each simulation was repeated twice, using different starting coordinates for each replica. These starting coordinates were obtained from preliminary runs and it was checked that they were structurally diverse and uncorrelated. Thus a total of 4 BEMD simulations were performed: two apo simulations (apoA and apoB) and two holo simulations (holoA and holoB).

Gaussian potentials of height 0.2 kJ.mol-1 were added every 2.0 ps. Collective variables and snapshots were saved every 2.0 ps and exchanges between replicas were attempted every 20.0 ps. All bond lengths were constrained to their equilibrium length with the LINCS algorithm. [76] The CVs used in the production runs were:

- **Apo simulations:** CV1: coordination number C_α_ atoms. width 0.7; CV2: coordination number C_γ_ atoms, width 0.5; CV3, similarity of backbone dihedral psi angle to α-helical region, width 0.25; CV4, correlation of successive backbone dihedral angles; CV5: number of backbone - backbone hydrogen bonds, width 0.25; CV6: number of sidechain - sidechain hydrogen bonds, width 0.25; CV7: number of sidechain - backbone hydrogen bonds, width 0.25;
- **Holo simulations:** CV1: coordination number C_α_ atoms. width 0.7; CV2: coordination number C_γ_ atoms, width 0.5; CV3, similarity of backbone dihedral psi angle to α-helical region, width 0.25; CV4, correlation of successive backbone dihedral angles; CV5: number of backbone - backbone hydrogen bonds, width 0.25; CV6: number of sidechain - sidechain hydrogen bonds, width 0.25; CV7: number of sidechain - backbone hydrogen bonds, width 0.25; CV8: minimum distance ligand C1 atom to peptide C_α_ atoms. C1 is the aromatic carbon atom bonded to the methylene group of **1**.

Accumulation of the biases gradually enables exploration of a larger range of values along each CV. This trend is more pronounced for CVs defined by counting interatomic contacts and eventually leads to the sampling of high energy configurations that cause hysteresis in the convergence of the free energy profiles for the biased replicas. However these high-energy configurations are almost never transferred to other replicas during replica exchange tests. To maintain a reasonable exchange rate between replicas and to focus conformational sampling in the regions of low free energy, half-harmonic potentials (walls) were added to penalize exploration of CV values below or above minimum/maximum values such that the computed free energy profiles are within approximately 10 k_B_T from the global minimum. The position of the walls was chosen by performing unrestrained preliminary BEMD runs.

- **Walls:** CV1: minimum 57, maximum 96; CV2: minimum 36, maximum 63; CV3: minimum 1, maximum 9; CV4, minimum 1.5, maximum 9.9; CV5, minimum 0.50, maximum 10.50; CV6, minimum 0.40, maximum 9.00; CV7 minimum 1.25, maximum 10.25; CV8 minimum 0.33, maximum 0.97.

With this setup the average exchange probability between biased replicas and neutral replicas was about 33% for both apo and holo simulations. The input files used to perform the apo and holo simulations are available in the supporting information (Dataset S1). On the basis of observed fluctuations in the values of the CVs over the duration of the BEMD simulations, the first 20 ns of the simulations was discarded to allow the Gaussian biases to compensate free energy barriers and enable broad sampling along each CV. The free energy profiles shown in Figure 2 and Figure 3 were taken as the negative of the averaged metadynamics biasing potential over the last 100 ns of each simulations.

Two different techniques were used to compute equilibrium properties from the BEMD ensembles. In the first approach, snapshots from all the biased replicas were reweighted using the method of Marinelli et al. [35] In this technique, the biased trajectories are first clustered in a N-dimensional CV space made of hypercubes forming a regular grid. The free energy of each bin is then estimated by a weighted histogram analysis procedure (WHAM) based on the number of snapshots and the value of the converged metadynamics bias potentials assigned to each bin. For the approach to be reliable, a large number of bins must be populated by several structurally similar snapshots. Increasing the dimensionality of the CV space decreases the statistics of each bin, but improves the structural similarity of snapshots within each bin. Multiple clustering schemes were tested to balance these two parameters, using the METAGUI plugin, [77] for the VMD software. [78] The best protocol identified for this system involved a 4-dimensional clustering using CV1, CV3, CV4, CV5 with a bin width of approximately 2σ_i_, where σ_i_ is the Gaussian width of CVi. These 4 CVs were chosen as they were found to be the least correlated with each other, thus maximizing structural similarity of snapshots assigned to each bin. The bin width of 2σ_i_ is on the order of the resolution of the metadynamics free energy profiles. With this setup about 9000 bins contained at least 5 snapshots. Lower dimensionality clustering produced bins that lumped together structurally dissimilar states, whereas higher dimensionality clustering yielded very few bins populated with more than five snapshots. Molecular observables were averaged between snapshots assigned to the same bin. Ensemble properties were then obtained by weighting the properties of each bin by its WHAM derived free energy. In the second technique, ensemble properties were computed by simple averaging of the properties of each snapshot recorded in the simulation of the neutral replica.

Two unbiased molecular dynamics simulations of c-Myc_402–412_ were also performed for comparison with the BEMD simulations (mdA and mdB). The simulations parameters were identical to the BEMD simulations, with the exception of the time step that was set to 5 fs as virtual sites were used, [79] and the simulations duration was 110 ns. The first 10 ns were discarded to enable relaxation of the system To evaluate statistical errors for the various computed properties from all simulations, standard errors were estimated from properties computed from two independent simulations.

### Simulations Analysis

To assess the accuracy of the computed structural ensembles, the software Camshift was used to predict experimentally measured backbone H, H_α_, C_α_ and C_β_ chemical shifts for c-Myc_402–412_. [39] Camshift does not predict chemical shifts for N and C terminal residues so no predictions for Tyr_402_ and Lys_412_ could be made. Secondary structure preferences were computed using several algorithms. DSSP, [40] STRIDE, [41] and PROSS. [42] The webserver δ2D was used to estimate secondary structure preferences from the measured chemical shifts. [43] For the BEMD simulations, the probability of contacts between protons in different peptide residues or the ligand was computed for the apo and holo ensembles and expressed as a contact matrix. A cutoff of 3 Å was used to define a proton-proton contact, which is intermediate between distances compatible with strong/medium NOEs. Small variations in this cutoff (±0.5Å) did not affect significantly the observed trends. RMSD clustering was performed using the method of Daura et al. to identify structurally distinct clusters of protein conformations and to estimate their population. In this approach, the RMSD of atom positions between all pairs of structures in a trajectory is first determined. For each structure, the number of structures that have a RMSD below a cutoff value are counted. The structure with the highest number of neighboring structures defines a cluster centre. This structure, along with all neighboring structures, is removed from the trajectory. This procedure is iterated until no structures are left unassigned. An advantage of this algorithm over alternative methods such as k-means or k-medoids is that the number of clusters is automatically determined, at the cost of a high memory requirement. [44] To reduce the memory requirements of the algorithm, a subset of the biased snapshots was used in the clustering analysis, by only selecting snapshots from bins that were within 6kT from the bin of lowest free energy. To estimate errors on the cluster populations, the ensembles from the two apo/holo simulations were combined. A RMSD cutoff of 3.5 Å was used to group structures. For the apo ensemble the RMSD calculations were performed using the coordinates of heavy atoms, excluding atoms that can form symmetry equivalent conformations (e.g. Valine C_γ_ atoms). For the holo ensemble, a different protocol was used to finely resolve different binding modes of the ligand. The RMSD calculations were performed on the protein C_α_ and C_β_ atoms and non-symmetry equivalent ligand heavy atoms. The ligand coordinates were weighted by a factor of 3 in the RMSD calculations to in order to cluster together conformations that contained similar ligand coordinates.

## Supporting Information

Figure S1 **Secondary structure content of c-Myc_402–412_.** Residue secondary structure preferences colored according to the STRIDE code (white: coil, cyan: turn, blue: 3_10_ helix, purple: α-helix, maroon: bend, yellow: extended). A) and B) BEMD ensembles from the neutral replicas for simulations apoA and apoB. C) and D) Unbiased ensembles from MD simulations mdA and mdB.
(TIF)

Figure S2 **Comparison of computed and observed secondary chemical shifts for apo c-Myc_402–412_.** A) ^1^H_α_ chemical shifts. B) ^13^C_α_ chemical shifts. C) ^1^H backbone amide chemical shifts. D) ^13^C_β_ chemical shifts. Black: experimental data. Red: predicted from MD simulation mdA. Blue: predicted from MD simulation mdB.
(TIF)

Figure S3 **Comparison of free energy profiles of c-Myc_402–412_ obtained from the neutral replica and the biased replicas.** Black: Neutral replica, Red: Biased replica. Data generated using BEMD simulation apoA.
(TIF)

Figure S4 **Hydrophobicity plot of the sequence of the c-Myc and Max bHLHZip domains.** Black: c-Myc. Red: Max. Regions with a positive score are considered hydrophobic. The location of the c-Myc segment corresponding to amino acids 401 to 406 has been highlighted in bold. Plots generated using a Kyte-Doolittle hydrophobicity scale. [80] To detect relatively short sequences of hydrophobic and aromatic sites that may interact favorably with small organic molecules the scale was modified so that Tyrosine has a hydrophobicity score equal to Phenylalanine and a window width of 3 was used. Plots produced using the sequences c-Myc_353–437_ (84 amino acids) and Max_24–102_ (78 amino acids).
(TIF)

Dataset S1 **Input files for the apo and holo BEMD simulations.**
(ZIP)
