# Supplementary figures and images for: The Impact of Small Molecule Binding on the Energy Landscape of the Intrinsically Disordered Protein C-Myc
Source: PLoS One. 2012 Jul 16;7(7):e41070. doi: 10.1371/journal.pone.0041070 (PMC3397933; doi:10.1371/journal.pone.0041070)

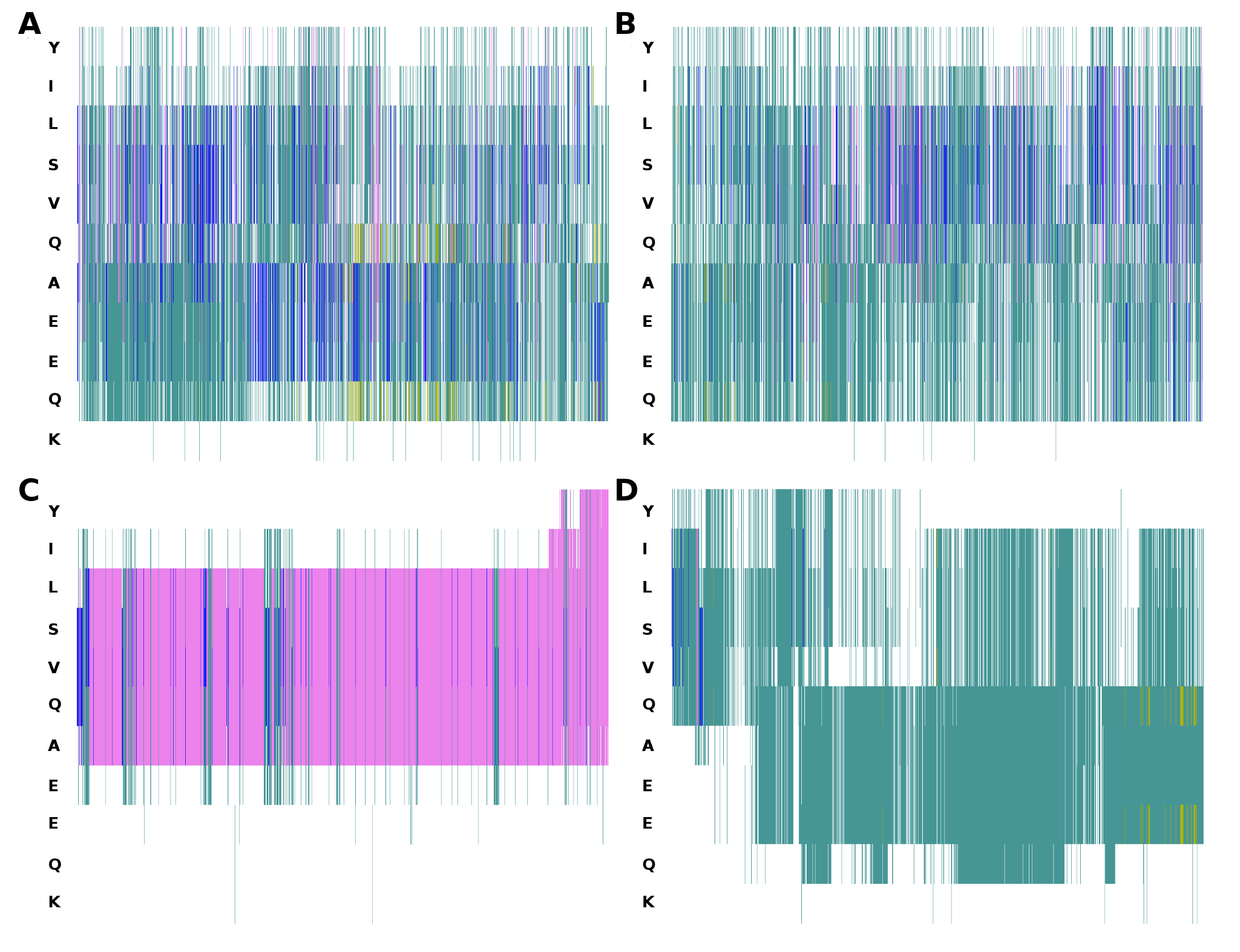

Supplement: Figure S1 — Secondary structure content of c-Myc402–412. Residue secondary structure preferences colored according to the STRIDE code (white: coil, cyan: turn, blue: 310 helix, purple: α-helix, maroon: bend, yellow: extended). A) and B) BEMD ensembles from the neutral replicas for simulations apoA and apoB. C) and D) Unbiased ensembles from MD simulations mdA and mdB. (TIF) [file pone.0041070.s001.tif]

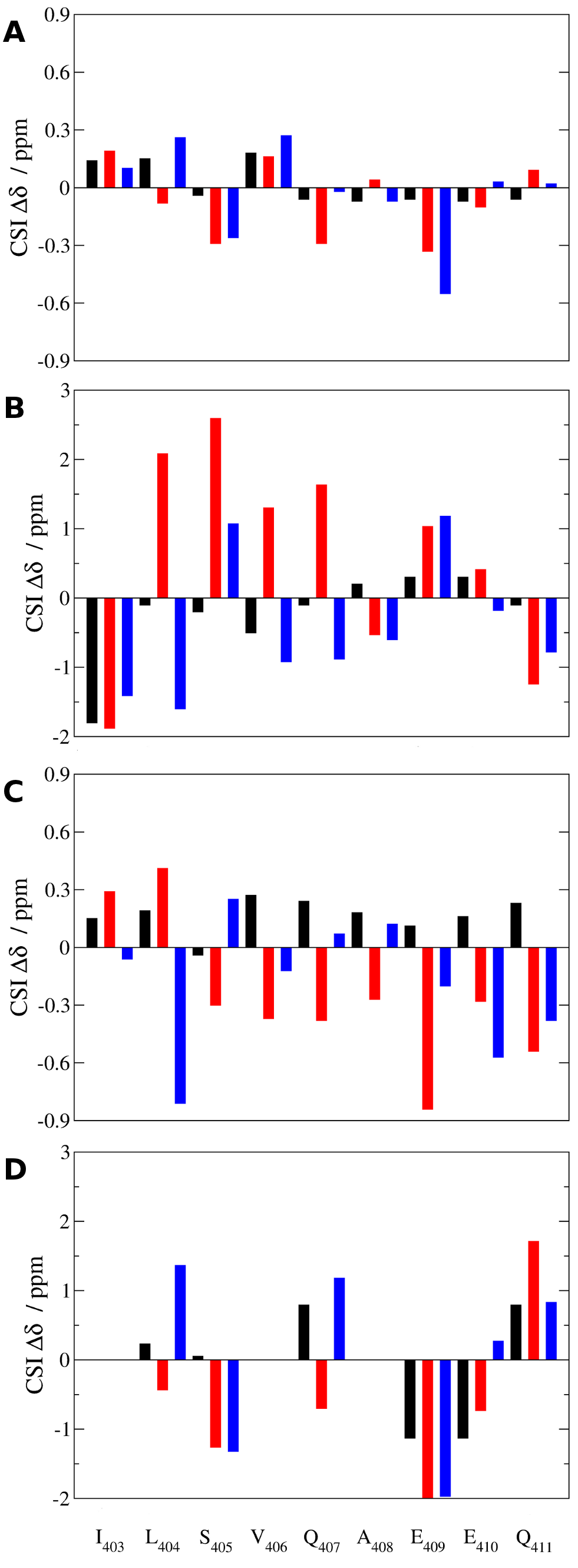

Supplement: Figure S2 — Comparison of computed and observed secondary chemical shifts for apo c-Myc402–412. A) 1Hα chemical shifts. B) 13Cα chemical shifts. C) 1H backbone amide chemical shifts. D) 13Cβ chemical shifts. Black: experimental data. Red: predicted from MD simulation mdA. Blue: predicted from MD simulation mdB. (TIF) [file pone.0041070.s002.tif]

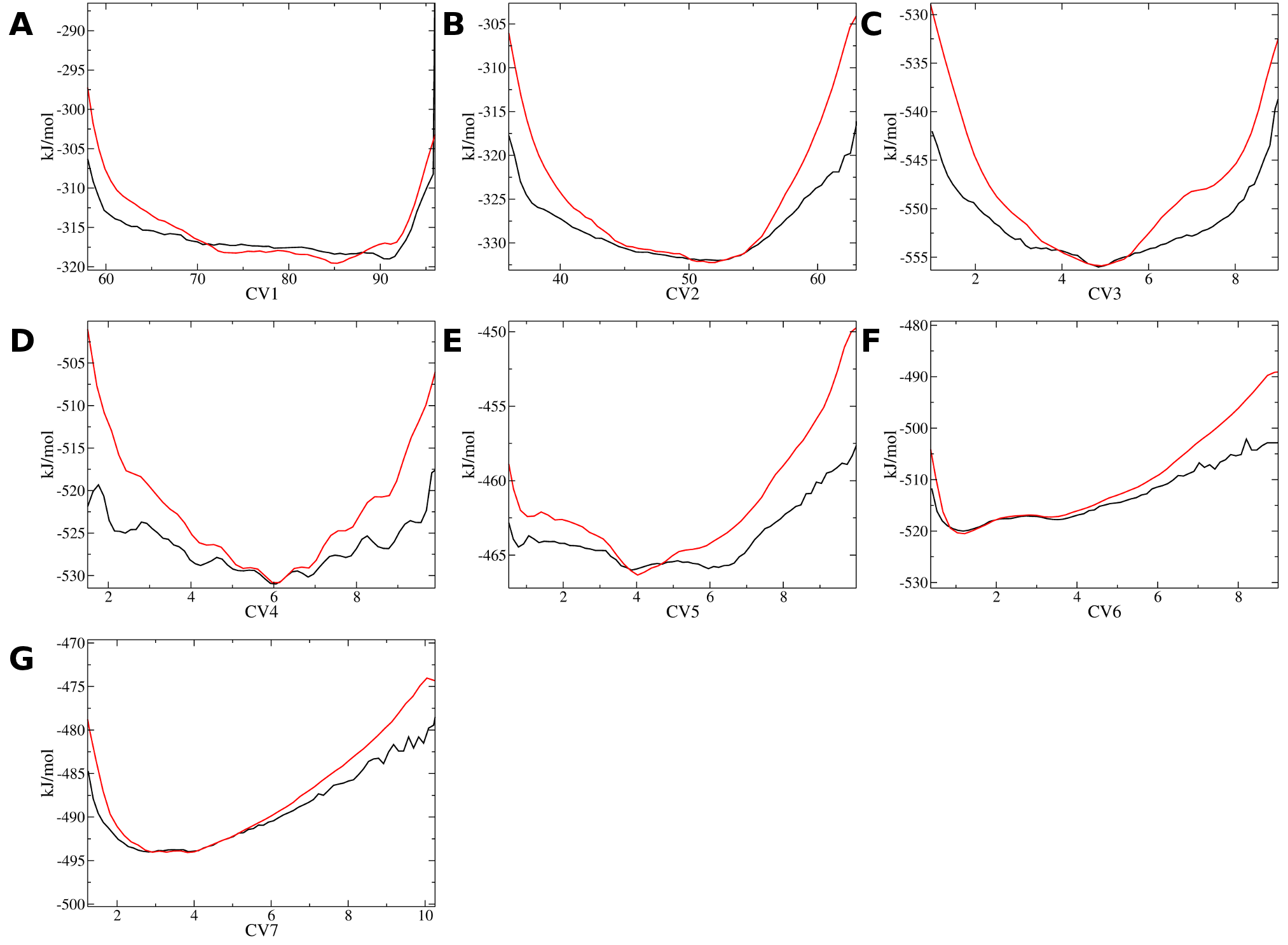

Supplement: Figure S3 — Comparison of free energy profiles of c-Myc402–412 obtained from the neutral replica and the biased replicas. Black: Neutral replica, Red: Biased replica. Data generated using BEMD simulation apoA. (TIF) [file pone.0041070.s003.tif]

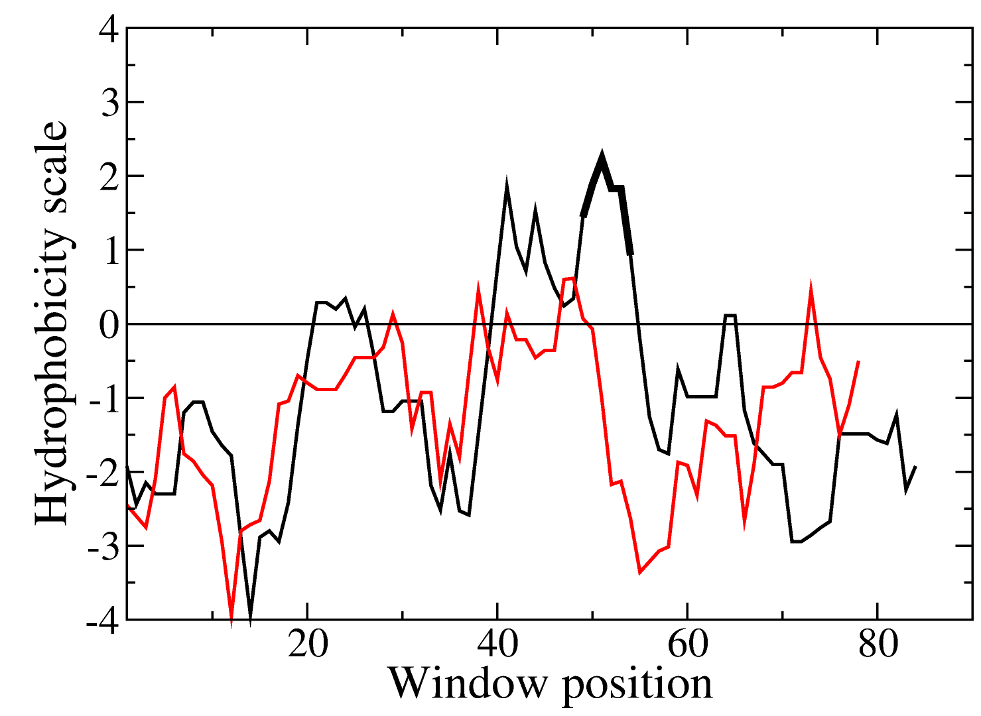

Supplement: Figure S4 — Hydrophobicity plot of the sequence of the c-Myc and Max bHLHZip domains. Black: c-Myc. Red: Max. Regions with a positive score are considered hydrophobic. The location of the c-Myc segment corresponding to amino acids 401 to 406 has been highlighted in bold. Plots generated using a Kyte-Doolittle hydrophobicity scale. [80] To detect relatively short sequences of hydrophobic and aromatic sites that may interact favorably with small organic molecules the scale was modified so that Tyrosine has a hydrophobicity score equal to Phenylalanine and a window width of 3 was used. Plots produced using the sequences c-Myc353–437 (84 amino acids) and Max24–102 (78 amino acids). (TIF) [file pone.0041070.s004.tif]
